# Unveiling the Genetic Diversity and Demographic History of *Coffea stenophylla* in Sierra Leone Using Genotyping-By-Sequencing

**DOI:** 10.3390/plants14010050

**Published:** 2024-12-27

**Authors:** Paul M. Lahai, Peter O. Aikpokpodion, Alieu Mohamed Bah, Mohamed T. Lahai, Lyndel W. Meinhardt, Seunghyun Lim, Ezekiel Ahn, Dapeng Zhang, Sunchung Park

**Affiliations:** 1 Sierra Leone Agricultural Research Institute (SLARI), IDA, Kenema 42215, Sierra Leone; paulmusalahai@yahoo.com; 2 Department of Crops Science, Faculty of Agriculture, Njala University, Mokonde 42215, Sierra Leone; 3 Department of Genetics and Biotechnology Faculty of Biological Sciences, University of Calabar, PMB 1115, Calabar 540271, Cross River State, Nigeria; paikpokpodion@gmail.com; 4 Department of Crop Science, Faculty of Agriculture, Eastern Technical University, Kenema 42215, Sierra Leone; profmtlahai@gmail.com; 5 Sustainable Perennial Crops Laboratory, United States Department of Agriculture, Agriculture Research Service, Beltsville, MD 2005, USA; lyndel.meinhardt@usda.gov (L.W.M.); seunghyun.lim@usda.gov (S.L.); ezekiel.ahn@usda.gov (E.A.)

**Keywords:** climate change, conservation, crop wild relatives (CWRs), isolate-breaking effect, genetic diversity, specialty coffee, West Africa

## Abstract

*Coffea stenophylla* is a rare Coffea species boasting a flavor profile comparable to Arabica coffee (*Coffea arabica*) and has a good adaptability to lowland tropical climates. This species faces increasing threats from climate change, deforestation, and habitat fragmentation in its West African homeland. Using 1037 novel SNP markers derived from Genotyping-by-Sequencing (GBS), we revealed the presence of three distinct natural populations (mean Fst = 0.176) in Sierra Leone. Evidence of recent bottlenecks and small effective population size (118–140) was found across all three populations, reflecting the impact of recent anthropogenic disturbances on this species. Using a model-flexible inference approach, we unveiled a strong ancient bottleneck approximately 23,000 years ago, coinciding with the last glacial maximum (LGM), followed by post-glacial expansion and divergence into distinct genetic clusters. A comparative analysis between ex situ genebanks and natural populations detected a significant gap in genetic diversity, with two out of three natural populations missing from the ex situ genebank collection. These findings highlight the urgent need to improve conservation practices for *C. stenophylla* in Sierra Leone. The novel SNP markers developed in this study provided valuable tools to support future efforts in conservation and utilization of *C. stenophylla* genetic resources in West Africa.

## 1. Introduction

Coffee, a globally beloved beverage, props up a multibillion-dollar industry and serves as the economic backbone of many tropical nations. Worldwide, an estimated 125 million people are dependent on coffee for their livelihoods [1,2]. In Sierra Leone, coffee used to be an important commodity, providing livelihood for thousands of smallholder farmers. Between the 1960s and 1980s, Sierra Leone produced around 20,000 tons of coffee annually. However, the 11-year civil war destroyed Sierra Leone’s coffee industry, leading to the abandonment of most coffee farms and a sharp decline in production. By 2022, coffee bean production had reduced to only 2580 tons [3]. A collaborative effort has been initiated to restore the coffee production capacity of Sierra Leone. However, the coffee supply chain continues to face a growing threat, such as climate change. Extreme weather events, including droughts, floods, and heatwaves, are disrupting harvests, jeopardizing crop yields, and putting the very future of the coffee industry at risk [4,5]. While sustainable practices are crucial for long-term viability and continued support for both the industry and coffee-dependent communities, progress towards implementing these practices remains slow [6,7].

The *Coffea* genus, belonging to the Rubiaceae family, comprises approximately 130 species [8,9,10]. Among them, Arabica (*Coffea arabica*) and robusta (*Coffea canephora*) are the two primary coffee crop species cultivated globally. Arabica coffee constitutes 56% of global production, while robusta accounts for 43%, with Liberica coffee (*Coffea liberica*) making up the remaining 1% [2]. Arabica coffee is believed to originate from the southwestern highland of Ethiopia, the Boma Plateau of South Sudan, and Mount Marsabit of Kenya [9,11]. The species are formed through natural interspecific hybridization between two diploid species, *C. canephora* and *C. eugenioides* [12]. This hybridization was followed by whole genome duplication, resulting in an allotetraploid (2n = 4x = 44) [13,14]. It is suggested that all Arabica plants grown worldwide originate from a single hybridization event, leading to limited initial genetic variation [15,16]. Furthermore, selective breeding during domestication has constrained the genetic pool, while self-pollination [17] in most Arabica varieties has further hindered the introduction of new genetic diversity. Due to its recent allotetraploid origin and strong bottlenecks during early cultivation and global dissemination, cultivated *C. arabica* today exhibits low genetic diversity [15]. Specifically, most prominent Arabica cultivars today descend from the Typica or Bourbon lineages. Genetic diversity increases in some cultivated genotypes, presumably because of introgressions deriving from the Timor hybrid [16].

Arabica coffee typically grows in highland regions (1000–2200 m), thriving in a cool-tropical climate with an optimum annual average temperature of 20–22 °C [18,19]. Due to its specific climatic requirements, the classic Bourbon-Typica lineages can only be cultivated successfully in a relatively narrow climate range. However, with the ongoing trend of global warming, Arabica faces challenges in sustaining its production. Despite its renowned superior flavor, the limited genetic diversity of Arabica poses obstacles to developing new cultivars necessary for ensuring the resilience of coffee production.

On the other hand, robusta coffee thrives predominantly in low-elevation regions (50–1500 m), naturally occurring across much of wet tropical Africa [20]. It is well-adapted to higher temperatures of 24–26 °C and exhibits higher productivity and caffeine content compared to Arabica [21,22]. It also demonstrates greater resilience to pests, diseases, and drought [18]. A spontaneous hybrid between *C. canephora* and *C. arabica* was discovered on the island of Timor in 1927, known as the Timor hybrid [13], exhibiting strong resistance to coffee leaf rust (*Hemileia vastatrix*) and increased tolerance to higher temperatures. Consequently, many modern cultivars incorporate genetic material from *C. canephora* through Timor hybrid-based introgression to improve disease resistance and temperature tolerance. Nevertheless, this breeding strategy has unintended consequences, including decreased beverage quality [23].

In addition to *C. arabica*, *C. canephora*, and *C. liberica*, several other coffee species are cultivated on a small scale, including *C. congensis*, *C. eugenioides*, *C. racemose*, *C. zanguebariae*, and *C. stenophylla* [9,10,24]. Among them, *C. stenophylla* is native to Guinea, Sierra Leone, and Ivory Coast [10,24]. Anecdotal and historical accounts suggest that this species has an excellent taste comparable to or even surpassing Arabica. Flourishing in its native lowland habitat (~400 m) in hot-tropical climates, *C. stenophylla* is recognized for its drought tolerance and partial resistance to coffee leaf rust. During the late 19th and early 20th centuries, *C. stenophylla* was cultivated in Sierra Leone and Guinea, to an extent where it was commercially exported to France. However, its cultivation dwindled thereafter, and widespread cultivation has not been pursued since then [24,25]. Recently, wild *C. stenophylla* plants were rediscovered in Sierra Leone, with their genomic and plastid DNA analyzed to confirm their identity [24], and a survey of morphological characteristics indicated the presence of genetic diversity among wild populations [26]. However, the species faces a restricted distribution with isolated populations and is currently under threat of extinction in the wild due to deforestation and agricultural activities [26,27,28]. Molecular characterization of *C. stenophylla* germplasm is urgently needed to support effective conservation and utilization of *C. stenophylla* genetic resources.

Genotyping-by-sequencing (GBS) has been widely used as a high-throughput and cost-effective method for genome-wide SNP discovery [29], proving successful in analyzing plant species with large genomes [30,31]. GBS offers several advantages over other genotyping methods, particularly when studying large sample sizes or seeking diverse genetic information. Its ability to generate thousands of SNPs simultaneously makes it efficient for large-scale studies, positioning GBS as a powerful and cost-effective tool for comprehensive genetic analyses.

The objectives of the present study are (1) to understand the genetic diversity and population structure in the *C. stenophylla* germplasm; (2) to reveal the demographic history of *C. stenophylla* natural populations; and (3) to assess potential gaps in genetic diversity within the ex situ genebank in Sierra Leone. To achieve these objectives, we characterized the genetic resources of *C. stenophylla* in Sierra Leone using SNP markers derived from GBS. We assessed the genetic diversity and population structure of natural populations and ex situ genebank collections of *C. stenophylla*. Additionally, we investigated the recent bottleneck and estimated the effective population size of *C. stenophylla* in Sierra Leone, where this species is considered vulnerable by the International Union for Conservation of Nature’s Red List of Threatened Species (IUCN Red List). These findings improved our understanding about the geographical structure of genetic diversity and the conservation status of this species. The SNP markers developed in this study and resultant information provide critical resources to address conservation and utilization challenges for *C. stenophylla* in West Africa and the international coffee community.

## 2. Materials and Methods

### 2.1. Plant Materials

**Figure 1:**
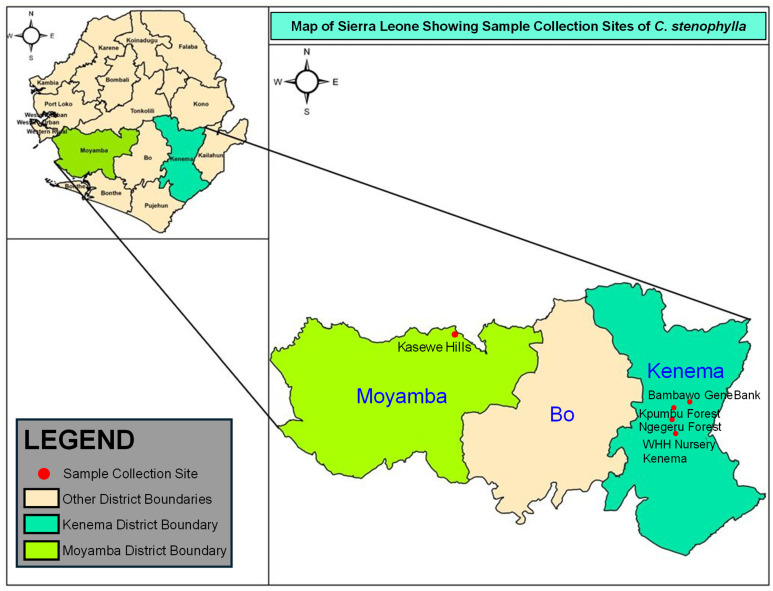
Geographic locations of the four sampled *C. stenophylla* populations from Sierra Leone, including three natural populations and one ex situ germplasm collection of the Sierra Leone Agricultural Institute (SLARI).

A total of 143 samples were collected from three natural populations and one ex situ germplasm bank in Kenema and Moyamba districts (notable areas for the existence of *C. stenophylla*). These sites are known to have hills that fall within the landscape of forest reserves (Figure 1; Table 1). Samples from Kenema district were collected from Kpumbu forest and Ngegeru forest. These two communities are situated 25 km and 10 km, respectively, southwest of Kenema city, the headquarters of the Eastern region. Other samples were collected from the Kasewe Hill Forest Reserve, about 32 km northwest of Moyamba town. The mean annual rainfall of Kambui and Kasewe forest reserves is 2546 and 2135 mm, respectively, with an average monthly temperature that ranges between 26 °C and 32 °C from June to October [30]. Both Kasewe and Kambui forests consist of terrain with steep and undulating slopes that reach an elevation of between 100 and 645 m above sea level. Kambui forest is divided into two distinct sections, i.e., Kambui north, which is about 20,348 ha, and Kambui south, with a land mass of about 880 ha [31]. This study basically focused on Kambui North, where the *C. stenophylla* had been discovered. The vegetation of these reserves is classified as evergreen with six months of continuous rain fall and a complex biodiversity that spans right across the untouched areas. The vegetation in the reserves has been classified as closed, consisting of three vegetation types: Albert logged forest (91.0%), farm bush (7.5%), and vine forest (0.7%) [31]. Kambui Hill Forest serves as protection for more than 12 catchments, and eight of these catchments currently supply water by gravity to the Kenema City and its environs [32], thus emphasizing its significance to not just biodiversity conservation but also its contribution to the sustenance of more than 200,000 inhabitants to the Kenema community. In addition to the natural populations, *C*. *stenophylla* samples were also collected from an ex situ collection established in Bambawo, an outstation of the Kenema Forestry and Tree Crops Research Centre (KFTCRC)—the tree crop sector of SLARI. Seedlings of wild *C. stenophylla* were planted at the Bambawo station in 2018 due to its nearness to the Kpumbu and Ngegeru communities, where natural forests of *C. stenophylla* are distributed (Figure 1).

A detailed list of all these accessions can be found in Table S1. From each coffee plant, one fully expanded young leaf was collected into labeled paper envelopes. A total of eight leaf disks were collected using the BioArk Leaf kit provided by LGC, Biosearch Technologies (https://www.biosearchtech.com/, accessed on 10 November 2024). The prepared BioArk Leaf kits were then submitted to LGC Genomics (Berlin, Germany) for DNA extraction and genotyping by sequencing.

### 2.2. Genotyping-By-Sequencing and SNP Identification

Genomic sequencing and SNP calling were conducted using the genotyping-by-sequencing (GBS) technology [33] in LGC genomics. In brief, DNA isolated from each sample was digested by the type II restriction endonuclease Ape KI. To distinguish samples, 5 to 8 bp barcode adaptors were added to the genomic DNA. Subsequently, DNA samples were subjected to 150 bp paired-end Illumina sequencing (http://www.illumina.com (accessed on 10 November 2024)). The raw reads were initially filtered based on the following criteria: (1) Reads were discarded if their 5′ ends did not match the restriction enzyme site; (2) reads that contained Ns were removed; (3) reads with a final length < 20 bp after trimming to achieve a minimum average Phred quality score of 20 over a window of ten bases were excluded. To generate reference clusters, reads were clustered using CD-HIT-EST v 4.6.1 [34], allowing up to a 5% difference. Singletons and clusters created from <100 reads were excluded from this process. Next, short reads were aligned to the reference cluster using Bowtie2 v2.4.2 [35]. Variants were called from the alignments (BAM files) using Freebayes v1.2.0 [36] with the following parameter settings: --min-base-quality 10, --min-supporting-allele-qsum 10, --read-mismatch-limit 3, --min-coverage 5, --no-indels, --min-alternatecount 4, --exclude-unobserved-genotypes, --genotype-qualities, --ploidy 2, --nomnps, --nocomplex, --mismatch-base-quality-threshold 10. The resulting SNPs were further filtered with the following criteria: (1) SNPs should be biallelic; (2) SNPs with a missing rate of >10% and minor allele frequency (MAF) of <5% were excluded; and (3) samples with missing SNP genotypes of >10% were also excluded. Finally, to obtain unlinked SNP markers, the GBS-derived SNP data were further filtered using the SNP and Variation Suite v 8.9.0 (Golden Helix Inc., Bozeman, Montana). SNPs with a pairwise linkage disequilibrium (LD) threshold of r^2^ ≥ 0.01 (with a window size of 50 and an increment of 5) were excluded from the dataset. The filtered data were then used for subsequent analyses of population genetics.

### 2.3. Genetic Diversity and Population Structure

To estimate genetic diversity within each population, we computed three metrics: (1) Expected heterozygosity (*H_exp_*) for all loci in each of the three natural populations and germplasm collection. (2) Observed heterozygosity (*H_obs_*), which measures the proportion of heterozygous individuals within a population at a specific locus, and (3) Pairwise relatedness (r) was computed based on maximum likelihood estimation of relatedness using the software ML-Relate (https://www.montana.edu/kalinowski/software/ml-relate/index.html (accessed on 10 November 2024)) [37]. Analysis of molecular variance (AMOVA, n = 999 permutations) and pairwise Fst were computed among the four sample sets using GenAlEx 6.501 [38,39] to determine the proportion of genetic variation attributable to differences between sampling sites. In addition, nucleotide diversity (π) was estimated using VCFtools v 0.1.15 [40].

To infer population structure, we used the STRUCTURE program version 2.3.4 [41] for Bayesian clustering analysis, employing an admixture model without prior information on genetic groups or geographic origins. Ten independent runs were assessed for each predefined number of clusters (K value) ranging from 1 to 10, with a burn-in of 50,000 iterations and 100,000 Markov chain Monte Carlo repetitions. The Delta K value was used to determine the optimal number of clusters that define the population [42,43] using Structure Selector, a web-based program. Individual samples were assigned to clusters based on the membership coefficients. Admixture proportions (Q values) were averaged across repetitions using CLUMPAK [44], and the proportion of ancestry was visualized using Structure Selector [45].

To further examine the genetic relationships among populations and germplasm groups, a neighbor-joining (NJ) clustering analysis was performed. Pairwise genetic distance [46] was calculated using the program MICROSATELLITE ANALYZER [47]. The NJ dendrogram was generated using PHYLIP v3.697 (https://evolution.genetics.washington.edu/phylip.html, accessed on 10 November 2024) and visualized using FigTree v1.4.3 (http://tree.bio.ed.ac.uk/software/figtree, accessed on 10 November 2024). In addition, we performed a Principal Coordinate Analysis (PCoA) using GenAlEx 6.501 [38,39]. The PCoA results are presented as two-axis PCO plots.

### 2.4. Recent Bottleneck and Reduction of Effective Population Size

To assess whether the *C. stenophylla* populations experienced recent genetic bottlenecks and reduction of effective population size, we used BOTTLENECK 1.2.02 [48] to evaluate any significant excess heterozygosity (*Ho* > *He*) under simulated mutation models. The Sign test and Standard difference tests were used under mutation-drift equilibrium. The analysis only included the three natural populations (Kasewe, Kpumbu, and Ngegeru), while the Bambawo_SLARI location was excluded because it is an ex situ germplasm collection propagated primarily from seeds collected from Ngegeru. Both tests were performed under the infinite allele mutation (IAM) model, the stepwise mutation model (SMM), and the two-phase model (TPM) with 1000 iterations. For the TPM model, the proportion of single stepwise mutation (SMM) was set to 70% and the variance was set to 30%. Additionally, we estimated the contemporary effective population size in the three natural populations using the linkage disequilibrium method [49], implemented in NeEstimator v2.1 [50]. A minimum allele frequency cut-off value of 0.05 was used, and a 95% confidence interval was obtained by jackknifing over individuals [51].

In addition to the SNP-based assessment on bottleneck and effective population size, we also inferred the demographic history of *C. stenophylla* directly using GBS data. The program Stairway plot 2 [52,53] was used to reconstruct the historical variation in population size, utilizing a multi-epoch model. A folded (ancestor allele unknown) site frequency spectrum was generated from the VCF file using easy SFS [54]. For this analysis, samples from the three natural populations (Kasewe, Kpumbu, and Ngegeru) were pooled, and the Bambawo_SLARI location was excluded. The generation time of *C. stenophylla* was set at five years, based on our observations at SLARI station. To account for potential variation in the generation time, a generation time of 10 years was also considered. We adopted the default mutation rate value in Stairway plot 2 for this analysis [52,53].

## 3. Results

### 3.1. SNP Identification and Population Genetic Analysis

The variant calling and data filtering resulted in an SNP data set of 115 accessions with 1037 unlinked SNPs, which has a missing rate less than 10%, MAF > 0.05, and pairwise linkage disequilibrium (LD) less than 0.01 (r^2^ ≤ 0.01). The 1037 SNPs were used in subsequent analyses of genetic diversity, population structure, and effective population size.

The descriptive statistics of genetic diversity, based on the 1037 unlinked SNP markers across the 115 *C. stenophylla* genotypes from four sites, are presented in Table 2. The mean observed heterozygosity (*H_o_*), expected heterozygosity (*H_e_*), and inbreeding coefficient (*F_IS_*) values were 0.290, 0.262, and −0.088, respectively. H_e_ ranged from 0.228 in Kasewe to 0.285 in Ngegeru, whereas Kpumbu (0.262) and Bambawo (0.281) fell in between. *H_o_* was nearly identical in Ngegeru (0.299), Kpumbu (0.307), and Bambawo (0.303), but slightly lower in Kasewe (0.258). Across all four sampling sites, *H_o_* consistently exceeded *H_e_*, leading to negative F_IS_ values (−0.132 to −0.050), indicating a slight deficit of homozygotes. Nonetheless, the F_IS_ values were close to zero, showing an overall balance between homozygotes and heterozygotes within populations. The average nucleotide diversity (π) of 0.123 was observed in *C. stenophylla* across the four sampling sites. Among them, Kasewe has the highest nucleotide diversity (0.158), followed by Kpumbu (0.121), Ngegeru (0.119), and Bambawo (0.095).

The mean relatedness values were also comparable across the four sampling locations. Bambawo_SLARI had the highest relatedness (0.0266), whereas Ngegeru had the lowest one (0.0168), but the difference was relatively small (Table 2). All four populations had relatedness values close to zero, meaning that individuals within each population were not closely related. The slightly higher relatedness in Bambawo_SLARI could be attributed to the fact that this site serves as ISNARs germplasm collection, which is mainly composed of individual plants derived from seeds collected from Ngegeru.

AMOVA was performed to analyze the partitioning of genetic variance among populations from different regions of *C. stenophylla*. The results indicated that most of the genetic variation (88.0%) occurred within regions, while 12.0% was attributed to variation between regions (Table 3). Genetic differentiation between regions was highly significant, as shown by Fst values (Table 4). The greatest differentiation was observed between Kpumbu and Kasewe forest reserve (0.227), while the lowest was between Bambawo_SLARI site and Ngegeru forest reserve (0.017). The significance of genetic differentiation between populations, except for the pair between Ngegeru and Bambawo_SLARI, was confirmed by a permutation test (Table 4).

**Figure 2:**
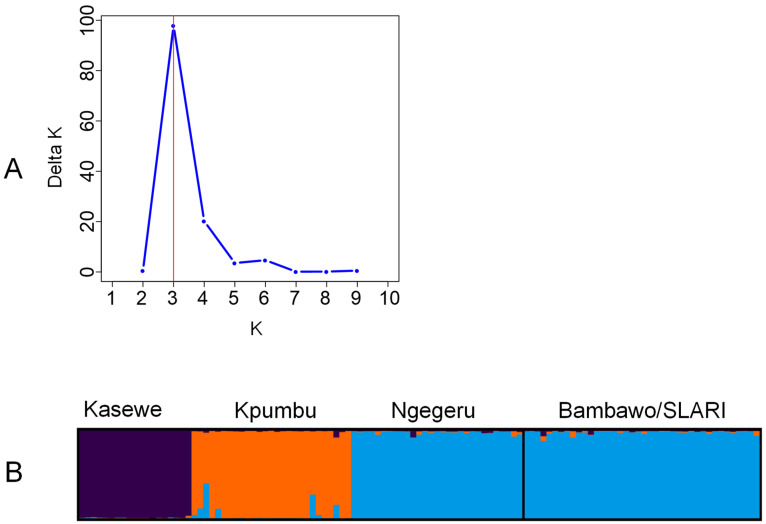
Bayesian clustering analysis of population structure in 115 *C. stenophylla* accessions. (**A**) Number of clusters based on the Evanno’s Delta K value. (**B**) Population structure of the 115 *C. stenophylla* germplasm accessions partitioned using Structure v2.3.4. Black vertical lines indicate the separation of the germplasm groups. Multiple colors within the genetic group imply admixed individuals under the scenario of K = 3. Geographical regions of sample’s origin are shown at the top.

The Bayesian clustering analysis using STRUCTURE revealed three ancestral groups (Figure 2), and the three groups corresponded to different geographical regions. Kpumbu and Kasewe forest reserves each formed a distinct group, while Ngegeru forest reserve and Bambawo_SLARI were grouped together. The clustering of Ngegeru and Bambawo SLARI can be explained by the fact that the germplasm in Bambawo_SLARI ex situ genebank was introduced from Ngegeru forest. Based on assignment probabilities (Q-value), we classified genotypes with a Q-value above 15% as having admixed ancestry. Overall, six hybrid genotypes were identified across the four locations: two in Kpumbu and two in Ngegeru, while no hybrids were found in Kasewe.

**Figure 3:**
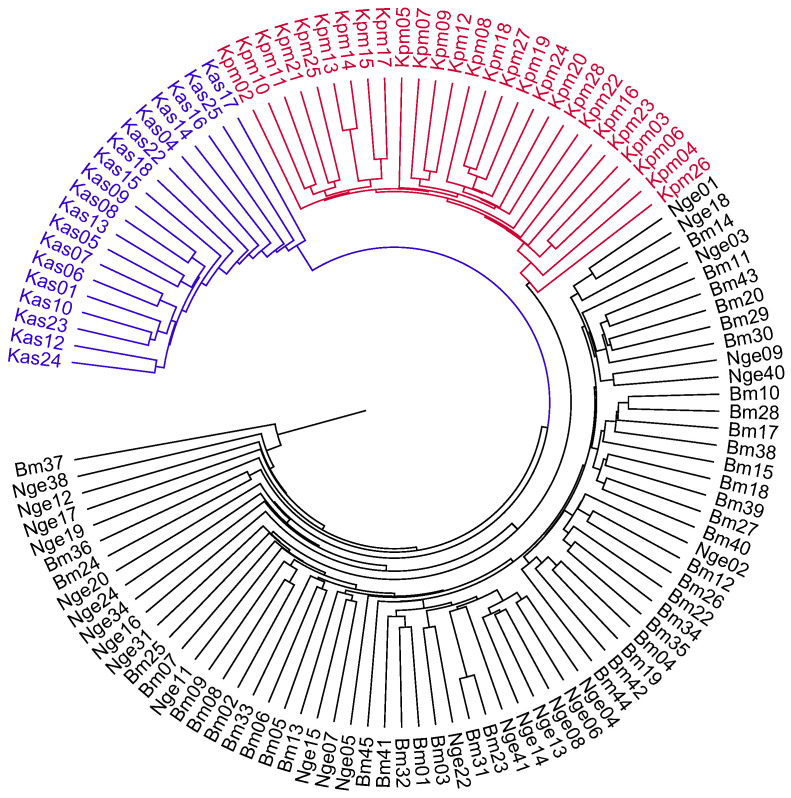
Neighbor-Joining tree of 115 *C. stenophylla* accessions based on 1037 SNP data for *C. stenophylla*. This tree was constructed based on genetic data from 115 coffee accessions. Branch colors represent the collection sites: blue for Kasewe, red for Kpumbu, and black for Ngegeru and Bambawo regions.

**Figure 4:**
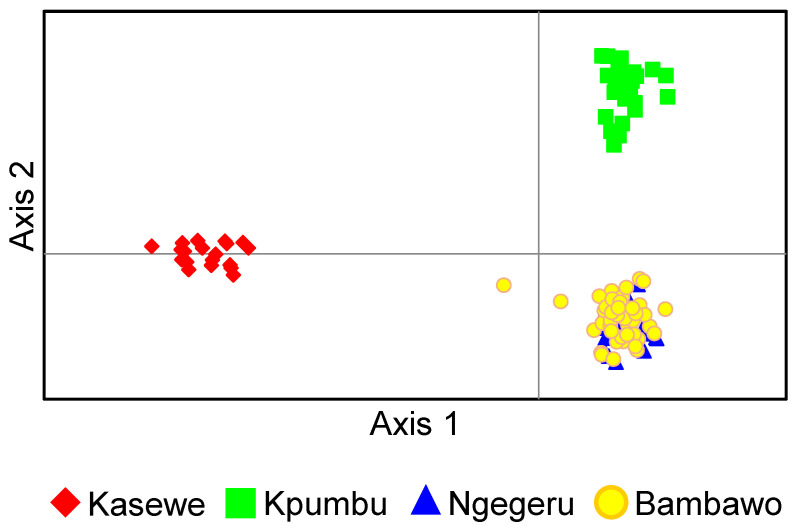
Principal coordinate analysis plot of 115 *C. stenophylla* genotypes in Sierra Leone based on 1037 SNP loci (Axis 1 vs. Axis 2). The three axes represent 38.5% of the total information (the first axis represents 20.1%, the second 13.8%, and the third 4.5%). The colored dots depict accessions belonging to Kasewe (Red), Kpumbu (Green), Ngegeru (Blue) and Bambawo (Yellow).

The results of STRUCTURE analysis were supported by distance-based ordination methods. The NJ tree showed that 115 genotypes of *C. stenophylla* could be grouped into three main clusters (Figure 3), which represent the samples from Kpumbu, Kasewe, Ngegeru, and Bambawo_SLARI (Figure 3). Similarly, the PCoA plot (Figure 4) also revealed three groups that largely corresponded to geographical origins (Figure 4). Consistently, in both the NJ tree and PCoA, accessions from Bambawo (the SLARI germplasm collection) and Ngegeru forest reserve showed considerable overlap, showing their high level of genetic similarity. Therefore, the result of multi-variant analysis suggested that the SLARI germplasm collection in Bambawo only represents one of the three main geographical clusters of *C. stenophylla*, highlighting a significant gap in genetic diversity within this collection.

### 3.2. Demographic History

The recent bottleneck analysis (excess heterozygosity) was performed using the computer program BOTTLENECK 1.2.02 under three different models: the infinite allele model (IAM), the 2-phase model (TPM), and stepwise mutation models (SMM) (Table 5). All three populations exhibited highly significant (*p* < 0.001) deviations from mutation-drift equilibrium, as shown by the Sign and Standard difference test, suggesting that each population has undergone a recent bottleneck event. Among the populations, Kasewe showed slightly less severe deviation than Kpumbu and Ngegeru under all three mutation models (Table 5).

Estimation of effective population size (*Ne*) was performed using NeEstimator v2. The result indicated moderate *Ne* values for all three populations: 140.4 for Kasewe, 126.6 for Kpumbu, and 117.8 for Ngegeru (Table 5).

**Figure 5:**
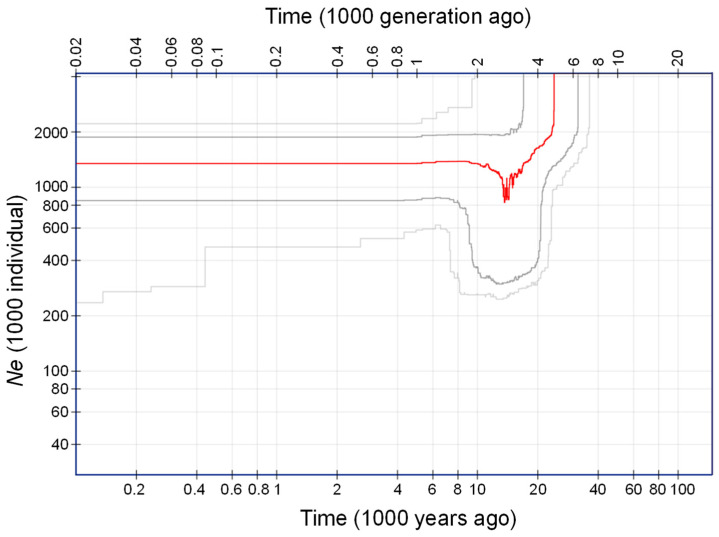
Demographic history of *C. stenophylla* inferred using Stairway plot 2, based on 72 pooled samples from the natural populations in Kasewe, Kpumbu, and Ngegeru. The generation time was set at five years. The solid red line represents the median estimation of effective population size, with the dark gray and light gray indicating the 75% and 95% confidence intervals, respectively.

Demographic history analysis using Stairway Plot 2 showed that *C. stenophylla* populations in Sierra Leone experienced a sharp decline in population size (Figure 5). The contraction started approximately 23,000 years ago, assuming an average reproductive maturity of five years for *C. stenophylla*. Therefore, the bottleneck event likely occurred during the Last Glacial Maximum (26,500–19,000 years ago). The decline led to the reduction of population size from an initial estimate of around 4000 to fewer than 1000, approximately 15,000 years ago (Figure 5). Following this reduction, the population of *C. stenophylla* expanded and stabilized about 10,000 years ago. These results indicated that *C. stenophylla* underwent a strong contraction during glacial periods, followed by post-glacial expansion and differentiation.

## 4. Discussion

### 4.1. Genetic Diversity in C. stenophylla Within and Between Geographical Regions in Sierra Leone

Understanding the genetic diversity and population dynamics of *C. stenophylla* is crucial for its conservation and utilization in breeding programs, particularly in the face of rapid global climate change. Recently, *C. stenophylla* has garnered significant attention due to its adaptability and agronomic potential, including its ability to withstand climate challenges and its superior flavor [10,24,26]. Unlike Arabica, which is a cool tropical crop, *C. stenophylla* can be cultivated in lower elevations where temperatures are typically higher, making it a promising candidate for adapting to changing climates [10,24,55]. However, *C. stenophylla* genetic resources are vulnerable due to extensive deforestation and habitat fragmentation in its native habitat in West Africa [24,55]. There is limited knowledge about its spatial pattern of diversity distribution, population structure, and evolution history, which has impeded effective conservation and breeding efforts. To address this gap, we conducted a comprehensive SNP-based genetic diversity analysis for *C. stenophylla*, using natural populations and genebank collections in Sierra Leone.

The genetic diversity, measured by gene diversity (*He*) and observed heterozygosity (*Ho*), is moderate and comparable across the three natural populations and the ex situ collection maintained by SLARI (Table 2). The Kasewe population in the south had slightly lower observed heterozygosity and gene diversity than the two natural populations in the east (Ngegeru and Kpumbu). However, the Kasewe population had a slightly higher nucleotide diversity (π = 0.158) than Ngegeru (π = 0.121) and Kpumbu (π = 0.119). Furthermore, the inbreeding coefficient (*F_IS_*) and relatedness are comparable among the three natural populations and the SLARI germplasm collection. The inbreeding coefficients in three natural populations are all negative but close to zero, indicating a balance between homozygotes and heterozygotes in these populations. These observations, together with the comparable effective population size in Kasewe (140.8), Ngegeru (126.6), and Kpumbu (117.8), allow us to conclude that there is a similar level of genetic diversity present within the south (Kasewe forest) and the east (Ngegeru and Kpumbu) populations.

Comparing our results to previous studies on wild coffee species is difficult due to the scarcity of literature on genetic diversity in wild coffee species. Nonetheless, as a diploid and outcrossing neo-tropical tree species, the gene diversity and observed heterozygosity found in this study (*He* = 0.225–0.278; *Ho* = 0.256–0.306) are substantially lower than those reported in *C. canephora* natural populations in Yangambi, DR Congo (*He* = 0.32; *Ho* = 0.34) [56]. It needs to be pointed out that *C. stenophylla* is widely distributed across Guinea, Ivory Coast, and Sierra Leone, whereas the present study only analyzed samples from Moyamba and Kenema districts within Sierra Leone, which may only represent a fraction of the whole primary gene pool of this species. For example, it is known that there are five populations of *C. stenophylla* in Ivory Coast [55]. Additional studies to include the full range of geographical distribution of this species are needed to gain a complete overview of genetic diversity in *C. stenophylla*.

Despite the fact that the samples only came from Sierra Leone, our result detected three distinct genetic clusters of *C. stenophylla*, as shown by STRUCTURE, Neighbor-Joining tree, and PCoA (Figure 2, Figure 3 and Figure 4). Nonetheless, the AMOVA results exhibited that the within-region variation is the dominant contributor, accounting for 88% of the total variation. In contrast, 12% of the total variation was explained by differences between populations. The large within-group variation indicates there is substantial gene flow among different populations, where individuals are readily exchanging genetic material. On the other hand, the large genetic variation within populations can be beneficial for the local population in terms of adaptability to changing environmental conditions. From the perspective of germplasm utilization, the large variation within population could have implications for differentiation of various phonotypic traits, such as flavor profiles and cupping quality, enabling selection of superior genotypes. From a crop breeding perspective, the substantial variation observed within populations is highly promising for establishing genetic improvement programs based on recurrent selection schemes.

The pairwise Fst values were moderately large, with the highest Fst observed in the Kasewe forest (average Fst = 0.200), followed by Kpumbu Forest (average Fst = 0.148), and Ngegeru forest (average Fst = 0.106). This West-East-oriented differentiation is consistent with the geographical features of Sierra Leone, where the country could be classified by different distinct geographical regions from the coast to the upland plateau and the eastern mountains. The Kasewe and the Ngegeru/Kpumbu communities are approximately 110 km apart (Figure 1) and have different climate conditions that potentially influence adaptive variation, physiological constraints, and phenotypic plasticity of *C. stenophylla*. It is noteworthy that although the Kpumbu and Ngegeru populations were sampled from the same district (Kenema District) and the two populations were from the same forest reserve, we detected two distinctive clusters with a moderate size of Fst (Fst = 0.110; *p* < 0.001). Population differentiation observed in this study may reflect the combined impact of decreased connectivity over geographic distance, climate change, and anthropogenic disturbance in the native habitat of this species, like many other outcrossing tree species in Neotropical conditions. For example, forest fragmentation contributes to population differentiation due to reduced gene flow between these fragmented areas, causing increased inbreeding and genetic drift [57,58]. Since there is little information on population genetics of *C. stenophylla*, it is difficult to assess the scale of changes in population differentiation between natural populations of this species. Nonetheless, the extent of population differentiation observed in this study is on a similar scale to different *C. canephora* populations in Uganda [59]. Based on 5,860 SNP markers derived from 323 candidate genes, there was a report of an average Fst of 0.142 among seven *C. canephora* populations, with the highest Fst of 0.267 between the two most differentiated populations [59]. Our result is also comparable to the findings in different *C. canephora* germplasm groups in Ghana [60], which observed an Fst of 0.256 in different germplasm groups based on the genotyping result of a low-density SNP array. Our result also indicates that knowledge gaps on outcrossing rate and reproductive biology in this species need to be filled through future investigations. These missing details will help to explain the observed results on the levels of heterozygosity, gene flow, and population differentiation.

### 4.2. Genetic Bottleneck and Estimation of Effective Population Size

The effective population size (*Ne*) is a critical parameter for understanding patterns of genetic variation in wild populations [49]. Our estimation, obtained using NeEstimator, indicates that the effective population size of *C. stenophylla* across the Kasewe, Kpumbu, and Ngegeru forests is between 117.8 and 140.4 (Table 5). These values are considered low relative to the thresholds generally suggested for conserving genetic diversity. According to [41] and the IUCN Red List’s Criterion thresholds, a *Ne* of at least 100 is required to limit loss in total fitness to ≤10%. Moreover, to ensure long-term evolutionary potential, a larger *Ne* (≥1000) is recommended to preserve fitness and adaptability over time [61].

The observed low *Ne* in this study is further supported by evidence of a genetic bottleneck (Table 4). Excess heterozygosity is a strong indication that a population undergoes a recent bottleneck. This is because the drastic reduction in population size will more likely cause the loss of rare alleles, which will result in a higher proportion of the remaining alleles being heterozygous [48]. All three natural populations (Kasewe, Kpumbu, and Ngegeru) exhibited highly significant (*p* < 0.001) deviation from mutation-drift equilibrium, as determined by the Sign and Standard difference tests. This suggests that *C. stenophylla* in Sierra Leone has undergone a recent bottleneck, likely within the past several dozen generations. Given that bottlenecks are typically detectable for only 40 to 80 generations [62], this event likely occurred within the past 400 years, assuming a 5-year generation time for *C. stenophylla*. Human-induced disturbances are likely responsible for this recent bottleneck.

It is important to note that deforestation in Sierra Leone has significantly accelerated in recent decades [63,64]. Sierra Leone lost 36% of its forests between 2002 and 2023, including 14% primary forests, due to farming activities, massive logging for timber, as well as urban expansion [64]. These anthropogenic disturbances have fragmented the landscape into small, isolated forest patches, which have severe impacts on genetic diversity and effective population size. Additionally, natural disasters may have reduced the number of reproductive trees, further limiting pollen donors and gene flow, leading to lower genetic diversity among the remaining populations. However, these impacts may not yet be fully reflected in the current population genetic diversity yet.

In addition to the SNP-based analysis, results from Stairway Plot 2 also suggest a significant reduction in the effective population size of *C. stenophylla* in Sierra Leone. Stairway Plot 2, a model flexible program based on a nonparametric method that does not require reference genomes or whole genome re-sequencing data, is particularly well-suited for non-modal organisms like *C. stenophylla* [53]. The analysis indicates that population contraction began approximately 23,000 years ago (Figure 5), coinciding with the growth of ice sheets in the southern hemisphere, which reached their maximum extent 26,500 years ago [65]. While West Africa was less directly affected by glaciation, the Last Glacial Maximum (LGM) saw a dramatic reduction in rainforest cover, with a few refugia surrounded by tropical grasslands [66]. The results of the demographic history analysis remained robust even when the generation time was adjusted from five to ten years (Figure S1). A similar timing of bottleneck events has been observed in other lowland tropical African tree species [67,68], suggesting that climatic changes during this period significantly influenced the evolutionary history of tropical forest trees in West Africa.

In summary, historical climate change appears to be a key factor driving the decline in the effective population size of *C. stenophylla* in Sierra Leone, with intensified human activities in recent years exacerbating the population decline. A much larger population size is necessary to maintain the genetic diversity in this species. *C. stenophylla* is threatened throughout its native ranges, particularly in Guinea [28]. It is currently listed as Vulnerable (VU) by the International Union for Conservation of Nature’s (IUCN) Red List of 2020 [69]. Our findings provided genetic evidence supporting this vulnerable status and clarified its conservation importance in Sierra Leone. Further systematic studies across multiple generations are needed to assess the full impacts of recent human activities on these populations.

### 4.3. Comparison of Genetic Diversity in Natural Populations and Ex Situ Collection of C. stenophylla

The natural populations of *C. stenophylla* are under threat from increasing rates of deforestation and habitat fragmentation. Ex situ conservation is an effective rescue measure for *C. stenophylla* by preserving genetic diversity outside of their natural habitat in controlled environments. Ex situ genebank allows for potential reintroduction into the wild if necessary and provides a backup for maintaining genetic diversity for future use [70,71]. A substantial number of germplasm accessions have been collected by SLARI and maintained in the ex situ genebank in Bambawo, Sierra Leone [63]. In addition to the SLARI collection, a nursery has been established in Pendembu by Welthungerhilfe (WHH) using the same source of wild population [63]. Since these ex situ collections were established based on seeds rather than vegetative clones, it allows us to compare the genetic diversity of true seedlings in the genebank with that of adult trees in natural populations. Our study showed that all the parameters of genetic diversity, including gene diversity, observed heterozygosity, inbreeding coefficient, relatedness, and nucleotide diversity, were comparable between the natural populations and the ex situ collection. This result suggested that seed harvesting is effective in terms of capturing allelic diversity from wild populations. However, the NJ tree, PCoA, and STRUCTURE analyses all showed that the *C. stenophylla* accessions in the ex situ genebank are overlapped and indistinguishable from the adult trees from Ngegeru forest, whereas accessions from the other two populations (Kasewe and Kpumbu) were almost completely missing. This result is not unexpected, as the SLARI collection was established using seeds collected exclusively from Ngegeru forest [63]. Our result, therefore, clearly demonstrated a major gap in genetic diversity represented in the SLARI ex situ collection. To ensure the capture of broad genetic variation in this species, accessions representing Kasewe, Kpumbu, and other existing populations in Sierra Leone need to be systematically collected and incorporated in the SLARI ex situ genebank. The wide range of genetic diversity also needs to be propagated in nurseries and distributed to farmers’ fields. Furthermore, the limitations of ex situ conservation need to be recognized, including the small sample size retained per accession, vulnerability to genetic drift, and lack of evolutionary process, which is particularly concerning in the context of accelerated climate change. In situ conservation will be needed to ensure the sustainable preservation of the *C. stenophylla* genetic resources. Specifically, innovative approaches should explore the potential for human engagement that benefits both livelihoods and biodiversity [4].

## 5. Conclusions

The present study revealed a significant genetic differentiation among three populations of *C. stenophylla* in Sierra Leone. We detected evidence of both ancient and recent bottlenecks, which led to a reduction in the effective population size of *C. stenophylla*. These population declines were likely driven by historical climate change during LGM and more recent anthropogenic activities in the last few hundred years. Considering the ecological and genetic risks, urgent conservation actions are needed to mitigate the vulnerability of this species. Establishing an ex situ genebank through seed samplings from different populations is an effective approach for preserving genetic diversity. To ensure maximum genetic diversity in the ex situ collection, systematic surveys of genetic diversity and representative sampling are required. Furthermore, in situ conservation will be needed to ensure sustainable preservation of *C. stenophylla* genetic resources.

## Figures and Tables

**Table 1:** Populations of *Coffea stenophylla* and their geographical locations in Sierra Leone.

Population	No. of Samples	Latitude	Longitude	Elevation (Masl *)
Kasewe	25	8°19′11.694″ N	−12°10′1.62″ W	416
Kpumbu	28	7°59′23.364″ N	−11°11′40.356″ W	375
Ngegeru	45	7°56′50.634″ N	−11°12′16.818″ W	466
Bambawo_SLARI	45	8°0′31.698″ N	−11° 8′8.496″ W	266
Total	143			

* masl: meters above sea level.

**Table 2:** Summary statistics of genetic diversity in three natural populations and one germplasm collection of *Coffea stenophylla* in Sierra Leone.

Locations	N	Ho	He	Inbreeding	Relatedness	Nucleotide
				Coefficient		Diversity
Kasewe	19	0.256	0.225	−0.103	0.0191	0.158
Kpumbu	27	0.306	0.260	−0.142	0.0236	0.121
Ngegeru	26	0.306	0.278	−0.076	0.0168	0.119
Bambawo_SLARI	43	0.294	0.272	−0.070	0.0266	0.095
Mean	28.8	0.291	0.259	−0.098	0.0215	0.123

**Table 3:** Analysis of Molecular Variance in three natural populations and one germplasm collection of *C. stenophylla* in Sierra Leone.

Source	df	SS	MS	Est. Var.	%	*p* Value
Among Populations	3	4516.4	1505.5	24.0	12.0%	<0.001
Within Populations	226	38,263.8	169.3	169.3	88.0%	
Total	229	42,780.2		193.3	100%	

**Table 4:** Pairwise Fst Value among geographical regions. All values were supported by significant *p*-values (<0.014), based on 999 permutations.

Locations	Kasewe	Kpumbu	Ngegeru	Bambawo (SLARI)
Kasewe				
Kpumbu	0.227			
Ngegeru	0.190	0.111		
Bambawo/SLARI	0.182	0.105	0.017	
Mean Fst	0.200	0.148	0.106	0.101

**Table 5:** Bottleneck and effective population size estimated for the three natural populations of *C. stenophylla* in Sierra Leone.

Mutation Models ^§^	Kasewe	Kpumbu	Ngegeru
IAM	0.0005 ***	0.0005 ***	0.004 ***
TPM	0.0001 ***	0.0001 ***	0.001 ***
SMM	0.001 ***	0.001 ***	0.005 ***
*Ne*	140.4	126.6	117.8

^§^ Bottleneck under three different models: (1) infinite allele (IAM), (2) two-phase model (TPM), and (3) stepwise mutation (SMM). Parameters for TPM: variance = 10%, proportion of SMM = 90%. P, probability. IAM, infinite allele model; TPM, 2-phase model; SMM, stepwise mutation model. *** indicates a significant deviation from equilibrium as a value less than 0.01.

## Data Availability

The GBS data are accessible in the Sequence Read Archive (SRA) under NCBI bioproject PRJNA1129264 (https://www.ncbi.nlm.nih.gov/bioproject (accessed on 10 November 2024).

## Supplementary Materials

The following supporting information can be downloaded at: https://www.mdpi.com/article/10.3390/plants14010050/s1, Figure S1: Demographic history of *C. stenophylla* inferred using Stairway plot 2, based on 72 pooled samples from the natural populations in Kasewe, Kpumbu, and Ngegeru. The generation time was set to 10 years. The solid red line represents the median estimation of effective population size, with the dark gray and light gray indicating the 75% and 95% confidence intervals, respectively. Table S1: List of 143 Coffea stenophylla trees collected from three natural populations and one ex situ germplasm collection in Sierra Leone.
