# Supplementary material for: Unveiling the Genetic Diversity and Demographic History of Coffea stenophylla in Sierra Leone Using Genotyping-By-Sequencing
Source: Plants (Basel). 2024 Dec 27;14(1):50. doi: 10.3390/plants14010050 (PMC11722797; doi:10.3390/plants14010050)
Supplement: Supplementary file 1 [file plants-14-00050-s001.zip › Supplimentary Figure S1.pdf]

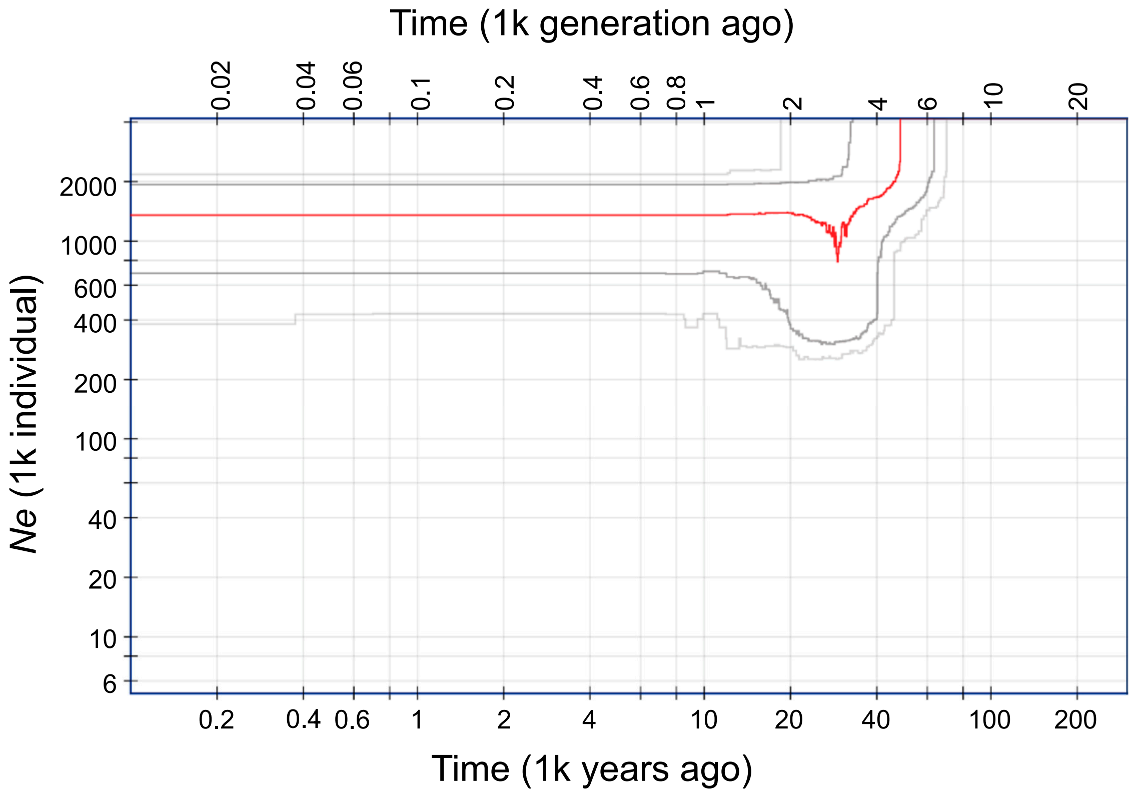


**Figure S1.** Demographic history of *C. stenophylla* inferred using Stairway plot 2, based on 72 pooled samples from the natural populations in Kasewe, Kpumbu and Ngegeru. The generation time was set to 10 years. The solid red line represents the median estimation of effective population size, with the dark gray and light gray indicating the 75% and 95% confidence intervals, respectively.
